# Host Plants Influence the Symbiont Diversity of Eriosomatinae (Hemiptera: Aphididae)

**DOI:** 10.3390/insects11040217

**Published:** 2020-04-01

**Authors:** Ting-Ting Xu, Li-Yun Jiang, Jing Chen, Ge-Xia Qiao

**Affiliations:** 1 Key Laboratory of Zoological Systematics and Evolution, Institute of Zoology, Chinese Academy of Sciences, Beijing 100101, China; xutingting@ioz.ac.cn (T.-T.X.); jiangliyun@ioz.ac.cn (L.-Y.J.); 2 College of Life Sciences, University of Chinese Academy of Sciences, Beijing 100049, China

**Keywords:** host plant, aphid relatedness, geographical distribution, gall, defensive symbiont

## Abstract

Eriosomatinae is a particular aphid group with typically heteroecious holocyclic life cycle, exhibiting strong primary host plant specialization and inducing galls on primary host plants. Aphids are frequently associated with bacterial symbionts, which can play fundamental roles in the ecology and evolution of their host aphids. However, the bacterial communities in Eriosomatinae are poorly known. In the present study, using high-throughput sequencing of the bacterial 16S ribosomal RNA gene, we surveyed the bacterial flora of eriosomatines and explored the associations between symbiont diversity and aphid relatedness, aphid host plant and geographical distribution. The microbiota of Eriosomatinae is dominated by the heritable primary endosymbiont *Buchnera* and several facultative symbionts. The primary endosymbiont *Buchnera* is expectedly the most abundant symbiont across all species. Six facultative symbionts were identified. *Regiella* was the most commonly identified facultative symbiont, and multiple infections of facultative symbionts were detected in the majority of the samples. Ordination analyses and statistical tests show that the symbiont community of aphids feeding on plants from the family Ulmaceae were distinguishable from aphids feeding on other host plants. Species in Eriosomatinae feeding on different plants are likely to carry different symbiont compositions. The symbiont distributions seem to be not related to taxonomic distance and geographical distance. Our findings suggest that host plants can affect symbiont maintenance, and will improve our understanding of the interactions between aphids, their symbionts and ecological conditions.

## 1. Introduction

Aphids have established sophisticated symbiotic associations with bacteria that contribute to their survival and environmental suitability. *Buchnera aphidicola*, which is the primary endosymbiont of aphids that occupies the specialized bacteriocytes, supplies essential nutrients to its host [1,2,3,4]. *B. aphidicola* is strictly maternally transmitted, and exhibits a pattern of codiversification with aphid hosts during long-term evolution [1,5,6,7,8,9,10,11,12,13].

Aphids also host various facultative symbionts, which are usually distributed randomly in aphids and undergo vertical and some horizontal transmission [14,15,16,17,18,19]. Facultative symbionts are generally not required for aphid development or reproduction, but infection with facultative symbionts can have context-dependent phenotypic effects on aphid hosts that influence major ecological processes, including defence against parasitic wasps [20,21,22,23,24,25] and fungal pathogens [26,27,28], protection against heat shock [29,30], interactions with host plants [31,32,33] and modification of body colour [34,35]. In addition, except for the primary endosymbiont *B. aphidicola*, some Lachninae species have established co-obligate endosymbiotic associations with *Erwinia haradaeae*, *Fukatsuia symbiotica*, *Hamiltonella defensa*, *Serratia symbiotica* and *Sodalis* sp. [36,37,38,39,40,41,42]. *Wolbachia* seems to be a co-obligate symbiont in *Pentalonia nigronervosa* Coquerel (Aphidinae: Macrosiphini) [43] (but see Manzano-Marín [44]).

The distributions of microbial symbionts differ across aphid species or populations, which may be influenced by both internal and external factors. The strong correlations between symbiont communities and host plants were revealed in *Acyrthosiphon pisum* (Harris) [45,46,47], *Aphis craccivora* Koch [48,49], *Aphis gossypii* Glover [50] and *Phylloxera notabilis* Pergande [51]. Studies have also found that aphid geographical distribution plays an important role in shaping symbiont microbiotas [52,53,54,55,56,57]. Furthermore, some other factors are suggested to be correlated with the symbiont distribution associated with aphids, such as host aphid species, plant diversity, parasitism rate and temperature [56,58,59,60]. Henry et al. [61] investigated the bacterial communities of 133 aphid species and highlighted the important roles of ecological conditions in structuring the symbiont distributions, whereas aphid phylogeny seemed to have no effect. McLean et al. [62] surveyed the microbiota of 46 aphid species and found that the microbiota composition was influenced by aphid relatedness rather than aphid ecology.

The associations between aphids and their bacterial symbionts are very complex, and a number of studies have been carried out to understand these associations, but little research has been performed in a specific aphid group to better illustrate the interactions with symbionts. Eriosomatinae is an extraordinary aphid group exhibiting very diverse life history traits and is widely distributed in the Holarctic and Oriental regions [63]. Most eriosomatine species show a heteroecious holocyclic life history, i.e., seasonal switching between primary and secondary host plants [64,65,66]. Eriosomatinae aphids have strong primary host specificity, with different patterns of host association among tribes. The three tribes, Eriosomatini, Fordini and Pemphigini (except for *Prociphilus*), are primarily associated with *Ulmus* and *Zelkova* (Ulmaceae), *Rhus* and *Pistacia* (Anacardiaceae) and *Populus* (Salicaceae), respectively. The secondary host plants, i.e., Cypeaceae, Graminaceae, Hypnaceae, Magnoliaceae, Pinaceae are more diverse. Eriosomatinae is also typically known for inducing galls on its primary host plants, secreting a visible wax coating and producing specialized sterile soldiers [67,68,69,70,71].

Although almost all viviparous aphid species harbour *B. aphidicola* as their primary endosymbiont, *Geopemphigus* Hille Ris Lambers (Eriosomatinae: Fordini) species have lost *B. aphidicola* and replaced it with the *Skilesia alterna* symbiont (phylum Bacteroidetes) [72]. Previous studies have shown that some Eriosomatinae species harbour four facultative symbionts, *Hamiltonella*, *Regiella*, *Serratia* and *Wolbachia* [16,61,73,74,75]. However, the detailed bacterial flora of this subfamily is still unclear.

In this study, using 16S rRNA Illumina sequencing data, we aimed to define the diversity of symbiont communities across members of subfamily Eriosomatinae by an in-depth survey and determine the interaction patterns and evolutionary forces that shape their composition and complexity.

## 2. Materials and Methods

### 2.1. Sampling and DNA Extraction

Thirty-four Eriosomatinae species belonging to eleven genera were sampled in this study, including 11 species within 3 genera in Eriosomatini, 3 species within 3 genera in Fordini, 20 species within 5 genera in Pemphigini (Table S1). Twenty-nine of these species are distributed in 11 provinces in China, 2 species in Ulan Bator (Mongolia) and 3 species in Illinois (USA); 3 species feed on Anacardiaceae, 1 species on Magnoliaceae, 2 species on Oleaceae, 3 species on Rosaceae, 15 on Salicaceae (*Populus*) and 9 on Ulmaceae (*Ulmus*). There was no information of host plant for sampled species *Colophina arctica* Zhang & Qiao. The main morphological characteristics used to identify the Eriosomatinae species included: the shape and distribution pattern of wax plates on dorsal body, the number and distribution pattern of dorsal setae, the number of segments of antennae, length in proportion of antennal segments, the shape, distribution and number of secondary rhinaria on antennal segments III–VI, siphunculi present or absent, the shape and position of galls, the shape of claw of hind tarsal segment, the venation of fore and hind wings and so on. We used the keys of Fauna Sinica (Eriosomatinae) [69], aphids on world’s plants from Blackman and Eastop (http://www.aphidsonworldsplants.info) and the revisions on some genera. Most keys were compiled based on alate viviparous females, but some samples used in present study only were collected fundatrix or apterous viviparous females, so we have no way to identify these samples to specific species. Therefore, we used sp. 1–n to distinguish unidentified different species in the same genus. Each species analysed was represented by 3–10 individuals from the same aphid species clone, which were then mixed together. All specimens were preserved in 95% and 75% ethanol for molecular experiments and voucher specimen collections, respectively. All aphid voucher specimens and samples were deposited in the National Zoological Museum of China, Institute of Zoology, Chinese Academy of Sciences, Beijing, China.

To remove body surface contaminations, aphid specimens were immersed in 70% ethanol, washed for 5 min (with vortex and centrifugation) and rinsed four times with sterile water. Total DNA was extracted from whole aphids using a DNeasy Blood and Tissue Kit (Qiagen, Hilden, Germany) following the manufacturer’s instructions, and two negative controls were set during DNA extraction. To verify the aphid species identification and eliminate parasitized aphids, the standard cytochrome oxidase subunit I (COI) barcode of each sample was amplified using the universal primer pair LCO1490 and HCO2198 [76].

### 2.2. Amplification and Sequencing of the 16S Ribosomal RNA Gene

Amplicons of the V3–V4 regions of the 16S ribosomal RNA (16S rRNA) gene were amplified using the primer pair 341F (5′-CCTAYGGGRBGCASCAG) and 806R (5′-GGACTACNNGGGTATCTAAT) with a barcode. All PCR amplifications were conducted in a 30 μL reaction mixture containing approximately 10 ng template DNA, 0.2 μM forward and reverse primers, 15 μL Phusion^®^ High-Fidelity PCR Master Mix (New England Biolabs, Massachusetts, United States) and double distilled water (ddH_2_O). The PCR conditions were set as follows: initial denaturation at 98 °C for 1 min, followed by 30 cycles of denaturation at 98 °C for 10 s, annealing at 50 °C for 30 s and elongation at 72 °C for 30 s and a final extension at 72 °C for 5 min. Each species was amplified in triplicate. PCR products were mixed in the same volume with 1× loading buffer, and bright bands corresponding to lengths between 400 and 450 bp were recovered using 2% agarose gel electrophoresis. All positive PCR products were mixed at equimolar ratio and then purified with a GeneJET Gel Extraction Kit (Thermo Fisher Scientific, Massachusetts, United States). A sequencing library was constructed using a NEBNext^®^ Ultra^TM^ DNA Library Prep Kit for Illumina (New England Biolabs, Massachusetts, United States) following the manufacturer’s recommendations, and index codes were added. Library quality was assessed with a Qubit^@^ 2.0 fluorometer (Thermo Fisher Scientific, Massachusetts, United States) and an Agilent Bioanalyzer 2100 system, and then the library pool was sequenced in paired-end 250 bp format using the Illumina HiSeq 2500 platform. The raw reads were deposited in the NCBI Sequence Read Archive (SRA) database under BioProject accession number PRJNA588521.

### 2.3. Sequencing Data Analysis

Raw reads were assigned to each sample based on their unique barcodes. Paired-end reads were merged using FLASH (v1.2.11) [77], and low-quality tags and chimaeras were filtered by QIIME with default settings [78]. The remaining sequences were clustered into operational taxonomic units (OTUs) at 97% identity by function *pick_de_novo_otus.py* in QIIME. The most abundant sequences of each OTU were chosen as the representative sequences and used to obtain taxonomic annotations with the RDP Classifier based on the SILVA 132 database [79,80,81]. Rare OTUs (<0.001% relative abundance in a given sample), chloroplast OTUs and OTUs identified as resulting from sequencing or PCR errors were excluded [82]. Finally, an OTU table containing the number of sequences per sample and taxonomic information was generated.

### 2.4. Symbiont Community Analysis

Analyses were executed with R software (v3.6.1) [83]. To mitigate the differences in sequencing effort, the number of sequences assigned to each sample was rarefied to the value (41236 reads) corresponding to the minimum sum of sequences across all the samples. Then, the relative abundance was calculated based on these rarefied abundance data by dividing the number of sequences per OTU by the total number of sequences for a given sample. Subsequent diversity analyses were performed based on these rarefied abundance data or relative abundance data.

To evaluate the within-sample diversity (alpha diversity) of the aphid bacterial community, the Shannon index and Simpson index of each species were calculated using the phyloseq package [84] based on the OTU abundance table. A rarefaction curve was generated based on the index of observed species.

Samples of Eriosomatinae were grouped according to host plant (Table 1). All samples were divided into four groups with a sample size of ≥3 (*n* = 30), and samples distributing in the same geography (Gansu Province, China) were divided into two groups with a sample size of ≥3 (*n* = 9). We performed analysis of variance (ANOVA) followed by Tukey’s honest significant difference (HSD) post hoc test using the vegan package [85] to determine pairwise differences in the alpha-diversity indices (Shannon index and Simpson index) across all groups. Differentiation of the bacterial communities between samples (beta diversity) was quantified by calculating Bray–Curtis dissimilarities using the vegan package [85]. The bacterial communities among groups were clustered using constrained principal coordinate analysis (CPCoA) and principal coordinate analysis (PCoA) based on the relative abundance of each genus and Bray–Curtis dissimilarities in the vegan package and ape packages, respectively [85,86], and plots were created by the ggplot2 package [87]. Based on the Bray–Curtis dissimilarities, permutational multivariate analysis of variance (PERMANOVA) was performed in the vegan package [85] to assess statistically significant differences among the host plant families. To compare the relative abundance of each symbiont between samples from different host plants, we conducted a Kruskal–Wallis test and pairwise comparisons, with a false discovery rate (FDR) *p*-value correction for multiple tests [88].

To explore the effect of aphid geographic distribution on structuring the microbial communities, we carried out correlation analysis between geographic distance and the beta-diversity index (Bray–Curtis dissimilarity). A geographic distance matrix was constructed from geographic points (latitudes and longitudes) using the function *GeoDistanceInMetresMatrix* written by Peter Rosenmai. Then, the Pearson correlation coefficient (*r*) between the two matrices was calculated, and the significance of the statistic was evaluated by a permutation procedure using the Mantel test in the vegan package [85]. We conducted the analysis using all samples (*n* = 34) and samples from the same plant family Salicaceae but distributing in different locations (*n* = 15), respectively.

To test whether more closely related aphid species have more similar microbial community, we measured aphid phylogenetic relatedness using the proportion of COI nucleotide sites at which two aphid species differ (p-distance). Then, we used Mantel test to assess the correlation between the p-distance matrix and the Bray–Curtis matrix [85].

All beta diversity analyses were conducted using all the bacterial genera, excluding primary endosymbiont *Buchnera*. We also divided the community into “known facultative symbionts” and re-ran all analyses to better determine the shaping factors of facultative symbiont.

Spearman’s rank correlation coefficients (*ρ*) were calculated for every pair of symbionts (primary endosymbiont and facultative symbionts) associated with Eriosomatinae to determine their interactions based on their relative abundances using the Hmisc package [89]. 

## 3. Results

### 3.1. Sequencing Data

Sequencing of the 16S rRNA V3–V4 amplicons yielded 8989997 raw reads. After quality filtering and removal of chimaeric sequences, a total of 5922611 effective tags with an average length of 429 nt were obtained. The sequences were classified into 931 OTUs at 97% sequence identity. The rarefaction curve for each sample tended to saturate (Figure S1).

### 3.2. Bacterial Diversity across Eriosomatinae Aphids

**Figure 1:**
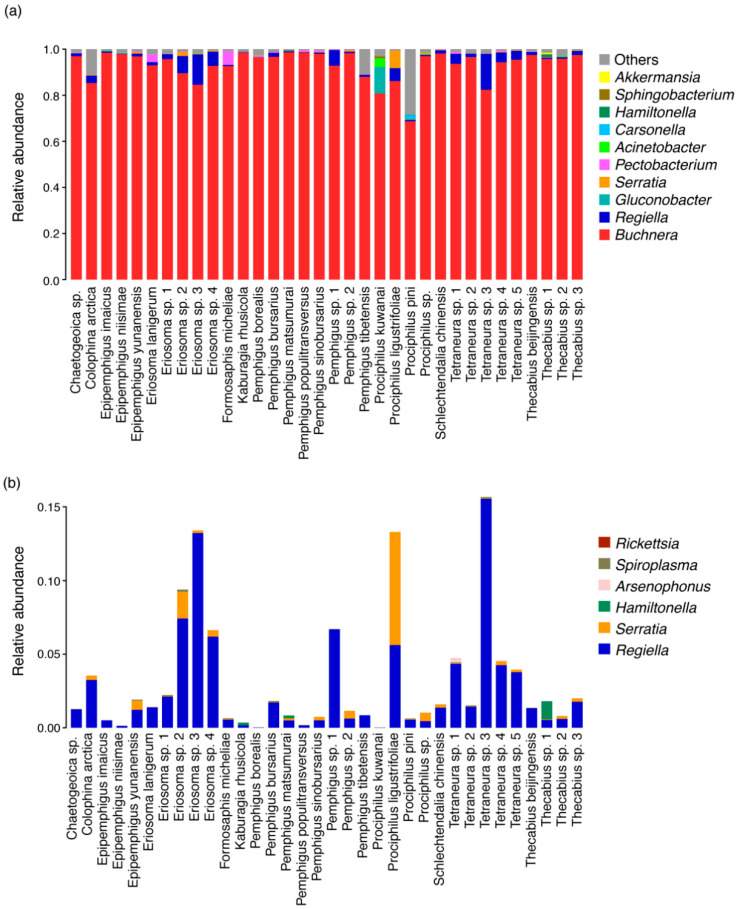
Bar plots of all bacteria (**a**) and facultative symbionts (**b**) associated with Eriosomatinae.

After discarding rare and chloroplast sequences and contaminations, 817 OTUs were obtained and annotated as belonging to 21 phyla, 180 families and 280 genera. Overall, 50.77% of these OTUs were attributed to Proteobacteria, 21.26% to Firmicutes and 11.47% to Actinobacteria. The alpha diversity of bacteria in Eriosomatinae was low (mean Shannon index = 0.29 and mean Simpson index = 0.12; Table S2). The bacterial community was dominated by *Buchnera*, *Regiella*, *Serratia*, *Hamiltonella*, the plant pathogen *Pectobacterium* and several environmental bacteria, such as *Gluconobacter* and *Acinetobacter* (Figure 1a and Table S3). The total relative abundance of aphid primary endosymbiont and facultative symbiont was greater than 96.00% in most samples and that of the other bacterial genera was less than 0.50%.

The primary endosymbiont *Buchnera* was found in all species, with an average relative abundance of 93.21%. A total of six aphid facultative symbionts were detected in Eriosomatinae aphids (Figure 1b and Table S4). *Regiella* inhabited all samples (detection frequency, 34/34; average relative abundance across all samples, 2.66%) and were thus the most common, followed by *Serratia* (33/34; 0.41%), *Hamiltonella* (17/34; 1.93%), *Arsenophonus* (12/34; 0.40%) and *Spiroplasma* (12/34; 0.13%). *Rickettsia* (5/34) was detected in Eriosomatinae species at very low abundance (<0.01%). Every facultative symbiont was represented by a small number of OTUs. There were five OTUs for *Regiella*, two for *Serratia* and only one for the remaining four symbionts. The numbers of reads belonging to the OTUs derived from the same genus were not equal, but in each sample, a single OTU from each genus was dominant. All of the samples contained at least two facultative symbionts (Table S5). The combination of *Regiella* and *Serratia* (7/34) was the most common, followed by those of *Hamiltonella*, *Regiella* and *Serratia* (6/34) and *Arsenophonus*, *Hamiltonella*, *Regiella* and *Serratia* (5/34).

### 3.3. Comparison of Microbiota Associated with Eriosomatinae among Plants, Geographic Distributions and Aphid Relatedness

Measurements of within-sample diversity (alpha diversity) of all detected bacteria or secondary symbionts showed no significant differences between eriosomatine species exploiting different host plants (*p* > 0.05; Tukey’s HSD test).

**Figure 2:**
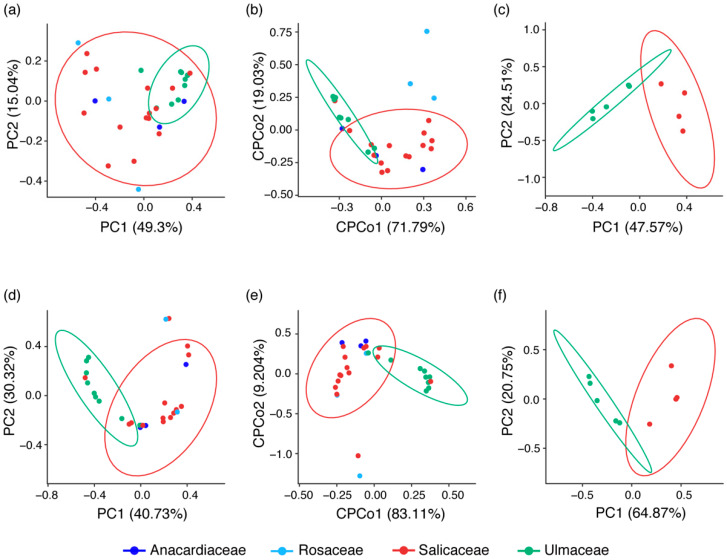
PCoA plot illustrating the separation of all samples from four host plant groups (**a** and **d**) and species distributing in the same location from two host plant groups (**c** and **f**) based on differences in bacterial community structure (**a** and **c**) and facultative symbiont community structure (**d** and **f**); CPCoA plot illustrating the separation of samples from four host plant groups based on differences in bacterial community structure (20.0% of the total variance and *p* = 0.003) (**b**) and facultative symbiont community structure (21.5% of the total variance and *p* = 0.001) (**e**). Colours correspond to different plant families, as shown in the legend. Ellipses cover 95% of the data for each plant families.

For the beta diversity, in PCoA analysis (Figure 2a), aphids from the same plant family tended to position near each other regardless of aphid species, and the PERMANOVA revealed significant differences in bacterial communities between host plant groups (*F*_3,26_ = 0.75, *R*_2_ = 0.25 and *p* = 0.01; Table 2a). CPCoA analysis revealed that the bacterial communities of aphids colonizing different plants tended to form distinct clusters (20% of the total variance and *p* = 0.003; Figure 2b). The bacterial communities of nine aphid species feeding on Salicaceae and Ulmaceae, which distributed in the same location (Gansu Province), are significantly different (*F*_1,7_ = 5.07, *R*_2_ = 0.42 and *p* = 0.008; Table 2a). PCoA analysis showed distinct clusters of nine aphid species feeding on the two plant families (Figure 2c). For comparison of the facultative symbiont community, PCoA and CPCoA (21.50% of the total variance and *p* = 0.001) analyses showed clear clusters of aphids feeding on the same plant family (Figure 2d,e). The PERMANOVA test (Table 2a) also revealed that facultative symbiont communities of aphids differed among species feeding on four host plants (*F*_3,26_ = 0.73, *R*_2_ = 0.27 and *p* = 0.002) and between nine species feeding on Salicaceae and Ulmaceae (*F*_1,7_ = 9.46, *R*_2_ = 0.57 and *p* = 0.013). PCoA analysis showed that nine aphid species feeding on the two plant families separated from each other (Figure 2f). Among all detected symbionts, significant food plant associations were detected for *Regiella* (*df* = 3, *χ*^2^ = 15.48 and *p* < 0.01). The relative abundance of *Regiella* in aphids feeding on Ulmaceae was significantly higher than that in aphids feeding on Anacardiaceae, Rosaceae and Salicaceae. 

In the Mantel test (Table 2b), we found no significant correlation between aphid geographic distances and Bray–Curtis dissimilarities of bacteria or facultative symbionts (*r* = −0.01–0.03 and *p* = 0.50–0.36). For 15 species feeding on the same plant genus (*Populus*), the aphid geographic distances were not significantly correlated with beta diversity of their bacteria or facultative symbionts (*r* = 0.14–0.14 and *p* = 0.19–0.20). 

The genetic distance (p-distance) and Bray–Curtis dissimilarities of both bacteria and facultative symbionts were not significantly correlated in Eriosomatinae (*r* = 0.07–0.01 and *p* = 0.16–0.41; Mantel test; Table 2b). For 15 species feeding on the same plant genus (*Populus*), the correlations between aphid genetic distance and Bray–Curtis dissimilarities of both bacteria and facultative symbionts were also not significant (*r* = −0.19–0.19 and *p* = 0.96–0.97; Table 2b).

### 3.4. Correlation Test between Different Symbionts Associated with Eriosomatinae Aphids

The resulting Spearman’s correlation coefficients are shown in Table S6. Significant positive correlations were found between *Arsenophonus* and *Hamiltonella* (*ρ* = 0.54 and *p* < 0.001) and between *Regiella* and *Serratia* (*ρ* = 0.40 and *p* < 0.05). *Buchnera* had both negative and positive correlations with facultative symbionts, showing a significant negative correlation with *Regiella* (*ρ* = −0.52 and *p* < 0.01) in particular. Except for these, there were no significant correlations between other symbionts.

## 4. Discussion

### 4.1. Diversity and Composition of Eriosomatinae Aphid Symbionts

Our study revealed that the microbiota associated with Eriosomatinae was dominated by a few bacterial taxa. Of the top 10 abundant genera, 4 were derived from genera that are known to be symbionts of aphids, including *Buchnera*, *Regiella*, *Serratia* and *Hamiltonella*. *Buchnera* inhabited all the sampled species, with the highest relative abundance (average relative abundance: 93.21%). The ubiquity and high abundance of *Buchnera* in the present study seem reasonable because of its obligate nutritive role and its long-term endosymbiotic association with aphids [1,2,3,5,6,7,8,90].

Six facultative symbionts were detected in Eriosomatinae, although their relative abundances were very low. These facultative symbionts exhibited various infection patterns. *Regiella* was detected in all the samples in the present study, but it was only detected in one of the 22 Eriosomatinae species by Russel et al. [16] using diagnostic PCR method and not detected in the 3 Eriosomatinae species by Henry et al. [61]. Most species sampled in these two studies were not included in our study.

The defensive role of *Hamiltonella*, *Rickettsia* and *Spiroplasma* has been documented in many studies [21,22,23,26,27,28,91,92]. However, in the present study, only a few samples harboured these symbionts, and they did so at extremely low abundance (<0.05%); this value was lower than that detected in Hormaphidinae (<1%) [93]. Most species in Eriosomatinae induce galls on their primary host plants or live in the roots of their secondary host plants [64,67,69,94,95,96,97,98]. Galls or living underground can provide protection against parasitoids and predators to the inducer aphid and its offspring [99,100,101,102], the biological role of which is similar to that of defensive symbionts. However, carrying protective symbionts incurs a cost in some aphid species, i.e., increasing development time, and reducing longevity and fecundity, which may lead to balancing selection on symbiont maintenance [29,91,103,104,105]. Hormaphidinae species, which can induce galls on their primary host plants but are primarily collected from secondary host plants, have a higher abundance of defensive symbionts and greater proportion of infected species than Eriosomatinae [93]. Compared with only wax protection, the life history trait of living in galls or plant roots provides stronger protection to Eriosomatinae against parasites and predators. Therefore, we suppose that the special life history traits possessed by Eriosomatinae reduce the pressure of natural enemies on these aphids, which may tip the balance against the infection frequency of defensive symbionts, as observed for ant tending [61].

### 4.2. Structure of Symbiont Community in Relation to Ecological Conditions

Several factors have been revealed in structuring the microbial profiles of aphids. Geographical distribution has been reported to influence symbiont communities of populations of *A. gossypii*, whereas host plants seem to have no impact [52,53,55,56,57]. However, based on extensive sampling efforts of *A. gossypii*, the study revealed that host plants rather than geographical distribution structured the symbiont community [50]. Strong associations between host plants and symbiont composition have been largely documented in pea aphid *A. pisum* and *A. craccivora* [45,46,47,48,49], whereas the role of geographical distribution in structuring aphid microbial communities has not been detected in these species. In addition, the study suggested that both geographic distribution and host plant influenced the structure and composition of the bacterial community of pea aphid [54]. Studies based on comprehensive sampling within family Aphididae revealed correlations between aphid symbiont, ant attending and host plant [61] and aphid relatedness [62]. 

However, most studies focused on different populations of one species. In the present study, we assessed the structuring factors of symbiont community of a specific aphid group, Eriosomatinae. Our results indicated that geographical distribution did not contribute to eriosomatine microbiota composition. For species feeding on the same plant species, the symbiont diversity was also not influenced by aphid locations. Aphid relatedness has no effect on the bacterial flora of Eriosomatinae, which was in accordance with the findings of Henry et al. [61].

In contrast to geographical distribution and species relatedness, host plant is an important variable explaining the symbiont community structure associated with Eriosomatinae. Ordination analyses showed species feeding on different plants have different symbiont communities. Statistical test revealed a prominent effect of host plant on bacterial communities. These findings provide a strong evidence that aphid host plants play an important role in symbiont distribution [46,49,61].

The symbiont distribution of aphid species feeding on Ulmaceae is unique. Symbiont communities of these species were clustered separately from those in other samples in ordination analyses. The Kruskal–Wallis test also revealed that the relative abundance of *Regiella* in samples from Ulmaceae were significantly higher than those in samples from other plants. These findings raise the possibility that certain facultative symbionts may be involved in the adaptation of aphids feeding on certain plants. Previous studies reported a role of facultative symbionts in aphid host plant use [31,32,33], but some found mixed results for symbiont importance in interactions between aphids and host plants [106,107]. Therefore, the interactions between symbionts associated with aphids and host plants remain to be experimentally tested. Regardless, our results demonstrated that host plants are among some of the forces that drive maintenance of facultative symbionts.

### 4.3. Interactions between Symbionts

The phenomenon of harbouring multiple facultative symbionts has been reported in aphids by several studies [46,54,59,108,109,110]. Similar to the findings of these studies, most eriosomatine species were superinfected with multiple facultative symbionts. Positive correlations between *Arsenophonus* and *Hamiltonella* and *Regiella* and *Serratia* were detected. Multiple-facultative symbiont infection may provide extra benefits to aphids. Coinfection of *Hamiltonella*–*Fukatsuia* and *Hamiltonella*–*Serratia* increased the resistance of *A. pisum* against parasites [111,112]. *A. gossypii* coinfected with *Hamiltonella* and *Arsenophonus* displayed enhanced fitness [113]. In contrast, Leclair et al. [114] revealed that coinfecting *Hamiltonella* negatively affected the beneficial phenotype provided by *Rickettsiella*. McLean et al. [115] revealed mixed results of multiple infections with different symbiont combinations. Furthermore, aphids hosting multiple symbionts may suffer additional costs [111,114]. These findings suggest that the interacting assemblage of facultative symbionts influences aphid fitness in different ways, which may be synergistic, additive or antagonistic.

Correlations were mostly absent between groups of symbionts, which suggested that the combinations of different symbionts were not specific. The relatively frequent superinfection with facultative symbionts in Eriosomatinae may be the result of horizontal transmission [54,108,116]. Eriosomatinae species are a typical heteroecious holocyclic aphid group [64,65]. Seasonal host alternation between primary and secondary host plants, migration between different secondary host plants and the presence of a sexual phase may greatly increase the possibility of horizontal transmission of facultative symbionts among Eriosomatinae species [116,117,118,119].

## 5. Conclusions

In conclusion, using Illumina sequencing of the 16S ribosomal RNA gene, we analysed the bacterial diversity in a particular aphid subfamily, Eriosomatinae. The microbiota of Eriosomatinae was dominated by heritable symbionts. The primary endosymbiont *Buchnera* unsurprisingly inhabited all species, in accordance with its obligate mutualist role. *Regiella* was the predominant facultative symbiont in the Eriosomatinae species. We found that symbiont diversity varied with host plant, suggesting an important role of the host plant in structuring the bacterial community associated with aphids. However, the aphid relatedness and geographical distribution seem to have no effect on Eriosomatinae symbiont composition. Moreover, combinations of multiple facultative symbionts were common in Eriosomatinae, but the interactions between them were very complex.

## Figures and Tables

**Table 1:** Grouping information for host plant of Eriosomatinae species.

Host Plant	Sample Number	Sample Name
Species across Eriosomatinae		
Anacardiaceae	3	*Chaetogeoica* sp., *Kaburagia rhusicola*, *Schlechtendalia chinensis*
Magnoliaceae	1	*Formosaphis micheliae*
Oleaceae	2	*Ligustrum lucidum*, *Prociphilus* sp.
Rosaceae	3	*Eriosoma lanigerum*, *Prociphilus kuwanai*, *Prociphilus pini*
Salicaceae	15	*Epipemphigus imaicus*, *Epipemphigus niisimae*, *Epipemphigus yunanensis*, *Pemphigus borealis*, *Pemphigus bursarius*, *Pemphigus matsumurai*, *Pemphigus populitransversus*, *Pemphigus sinobursarius*, *Pemphigus* sp. 1, *Pemphigus* sp. 2, *Pemphigus tibetensis*, *Thecabius beijingensis*, *Thecabius* sp. 1, *Thecabius* sp. 2, *Thecabius* sp. 3
Ulmaceae	9	*Eriosoma* sp. 1, *Eriosoma* sp. 2, *Eriosoma* sp. 3, *Eriosoma* sp. 4, *Tetraneura* sp. 1, *Tetraneura* sp. 2, *Tetraneura* sp. 3, *Tetraneura* sp. 4, *Tetraneura* sp. 5
Species from Gansu Province		
Salicaceae	4	*Pemphigus sinobursarius*, *Thecabius* sp. 1, *Thecabius* sp. 2, *Thecabius* sp. 3
Ulmaceae	5	*Tetraneura* sp. 3, *Tetraneura* sp. 4, *Tetraneura* sp. 5, *Eriosoma* sp. 2, *Eriosoma* sp. 3

**Table 2:** Statistical test results for bacterial and facultative symbiont communities in relation to different factors.

**(a)**				
	**Bacterial Community**		**Facultative Symbiont Community**	
Host plant	All 4 groups	2 groups	All 4 groups	2 groups
	0.25, **0.010**	0.42, **0.008**	0.27, **0.002**	0.57, **0.013**
**(b)**				
	**Bacterial Community**		**Facultative Symbiont Community**	
	All species	15 species	All species	15 species
Geographic distribution	−0.01, 0.50	0.14, 0.19	0.03, 0.36	0.14, 0.20
Aphid relatedness	0.07, 0.16	−0.19, 0.96	0.01, 0.41	−0.19, 0.97

**(a)**: Permutational multivariate analysis of variance (PERMANOVA) for the effects of host plant on bacterial and facultative symbiont diversity. The values in each cell represent *R*^2^ and *p*. **(b)**: Mantel test between aphid geographical distance and Bray–Curtis dissimilarity and between p-distance of aphid species and Bray–Curtis dissimilarity. The values in each cell represent *r* and *p*. Significant *p* values (*p* < 0.05) are in bold.

## Supplementary Materials

The following are available online at https://www.mdpi.com/2075-4450/11/4/217/s1, Figure S1: rarefaction curve for each sample of Eriosomatinae based on the index of observed species, Table S1: voucher information and GenBank accession numbers of reference COI sequences, Table S2: the Shannon index and Simpson index of Eriosomatinae bacterial community, Table S3: the relative abundance of top 10 bacterial genera in Eriosomatinae, Table S4: OTUs and reads number of facultative symbionts, Table S5: infection pattern of facultative symbionts in Eriosomatinae and Table S6: spearman correlation coefficients of symbionts in Eriosomatinae.
