# Supplementary material for: Host Plants Influence the Symbiont Diversity of Eriosomatinae (Hemiptera: Aphididae)
Source: Insects. 2020 Apr 1;11(4):217. doi: 10.3390/insects11040217 (PMC7240687; doi:10.3390/insects11040217)
Supplement: Supplementary file 1 [file insects-11-00217-s001.pdf]

Supporting information

Table S1. Voucher information and GenBank accession numbers of reference COI sequences.
Table S2. The Shannon index and Simpson index of Eriosomatinae bacterial community.
Table S3. The relative abundance of top ten bacterial genera in Eriosomatinae.
Table S4. OTUs and reads number of facultative symbionts.
Table S5. Infection pattern of facultative symbionts in Eriosomatinae.
Table S6. Spearman correlation coefficients of symbionts in Eriosomatinae.
Figure S1. Rarefaction curve for each sample of Eriosomatinae based on the index of observed species.

Table S1. Voucher information and GenBank accession numbers of reference COI sequences.

Species	Voucher number	Collection date	Host plant	Location	Longitude	Latitude	COI
**Eriosomatinae Eriosomatini**							
Colophina arctica	23540	1 Aug. 2005	Unknown	Beijing, China	40.47	116.99	JQ916891
Eriosoma lanigerum	15412	21 Aug. 2003	*Malus domestica* (Rosaceae)	Tibet, China	29.64	94.36	JQ916894
Eriosoma sp. 1	22383	22 Jun. 2009	*Ulmus pumila* (Ulmaceae)	Ulan Bator, Mongolia	47.00	107.00	JX536311
Eriosoma sp. 2	22920	24 May 2009	*Ulmus pumila* (Ulmaceae)	Gansu, China	35.88	104.16	JX536312
Eriosoma sp. 3	23044	2 Jun. 2009	*Ulmus pumila* (Ulmaceae)	Gansu, China	34.36	106.00	JX536316
Eriosoma sp. 4	23142	10 Jun. 2009	*Ulmus* sp. (Ulmaceae)	Sichuan, China	28.76	103.18	JX536318
Tetraneura sp. 1	22389	22 Jun. 2009	*Ulmus pumila* (Ulmaceae)	Ulan Bator, Mongolia	47.00	107.00	JQ916865
Tetraneura sp. 2	22400	4 Jul. 2009	*Ulmus pumila* (Ulmaceae)	Beijing, China	39.85	116.39	JQ916866
Tetraneura sp. 3	22926	24 May 2009	*Ulmus pumila* (Ulmaceae)	Gansu, China	35.88	104.16	JQ916872
Tetraneura sp. 4	23047	2 Jun. 2009	*Ulmus pumila* (Ulmaceae)	Gansu, China	34.36	106.00	JQ916881
Tetraneura sp. 5	23081	3 Jun. 2009	*Ulmus* sp. (Ulmaceae)	Gansu, China	34.43	105.96	JQ916885
**Eriosomatinae Fordini**							
Chaetogeoica sp.	15300	14 Jul. 2004	*Pistacia chinensis* (Anacardiaceae)	Shaanxi, China	34.50	107.83	JX536320
Kaburagia rhusicola	15699	26 Jun. 2004	*Rhus* sp. (Anacardiaceae)	Shaanxi, China	32.99	107.76	JQ916893
Schlechtendalia chinensis	15703	2 Sep. 2004	*Rhus chinensis* (Anacardiaceae)	Sichuan, China	29.52	103.33	JQ916860
**Eriosomatinae Pemphigini**							
Epipemphigus imaicus	25471	27 Jul. 2009	*Populus yunnanensis* (Salicaceae)	Yunnan, China	27.85	99.74	JQ916892
Epipemphigus niisimae	13509	1 Jul. 2002	*Populus* sp. (Salicaceae)	Tibet, China	29.81	93.84	KC710380
Epipemphigus yunnanensis	18234	27 Apr. 2006	*Populus yunnanensis* (Salicaceae)	Yunnan, China	26.83	100.27	JX627585
Formosaphis micheliae	18074	22 Oct. 2005	*Michelia maudiae* (Magnoliaceae)	Fujian, China	27.07	117.06	JQ916862
Pemphigus borealis	25483	29 Jul. 2010	*Populus yunnanensis* (Salicaceae)	Yunnan, China	27.01	100.26	JX536301
Pemphigus bursarius	17573	21 Jul. 2005	*Populus* sp. (Salicaceae)	Jilin, China	43.70	128.70	KC710390
Pemphigus matsumurai	23129	9 Jun. 2009	*Populus davidiana* (Salicaceae)	Sichuan, China	28.28	102.98	JQ916888
Pemphigus populitransversus	14166	13 Jun. 2001	*Populus* sp. (Salicaceae)	Illinois, USA	38.00	−89.15	KC710383
Pemphigus sinobursarius	22964	25 May 2009	*Populus cathayana* (Salicaceae)	Gansu, China	34.41	104.63	KC710395
Pemphigus sp. 1	14139	1 Jun. 2001	*Populus* sp. (Salicaceae)	Illinois, USA	40.00	−89.00	KF311113
Pemphigus sp. 2	14167	13 Jun. 2001	*Populus* sp. (Salicaceae)	Illinois, USA	38.00	−89.15	KF311114
Pemphigus tibetensis	15429	23 Aug. 2003	*Populus* sp. (Salicaceae)	Tibet, China	29.66	91.17	JQ916896
Prociphilus kuwanai	24365	9 May 2010	*Pyrus betulifolia* (Rosaceae)	Henan, China	32.76	113.50	JX536291
Prociphilus ligustrifoliae	22989	28 May 2009	*Ligustrum lucidum* (Oleaceae)	Gansu, China	33.55	104.84	JQ916876

Prociphilus pini	16169	13 May 2005	*Crataegus* sp. (Rosaceae)	Beijing, China	40.54	116.81	JQ916861
Prociphilus sp.	Y8936	3 Jul. 2010	*Fraxinus chinensis* (Oleaceae)	Hebei, China	38.68	116.08	JX536290
Thecabius beijingensis	15739	31 Jul. 2004	*Populus davidiana* (Salicaceae)	Heilongjiang, China	53.48	122.36	JX536307
Thecabius sp. 1	22912	23 May 2009	*Populus cathayana* (Salicaceae)	Gansu, China	35.82	104.08	JX536304
Thecabius sp. 2	22942	25 May 2009	*Populus cathayana* (Salicaceae)	Gansu, China	34.54	104.07	JX536305
Thecabius sp. 3	22956	25 May 2009	*Populus cathayana* (Salicaceae)	Gansu, China	34.54	104.07	JX536306

Table S2. The Shannon index and Simpson index of Eriosomatinae bacterial community.

Species	Shannon	Simpson
Chaetogeoica sp.	0.22	0.06
Colophina arctica	0.54	0.26
Epipemphigus imaicus	0.12	0.03
Epipemphigus niisimae	0.13	0.04
Epipemphigus yunnanensis	0.19	0.06
Eriosoma lanigerum	0.39	0.13
Eriosoma sp. 1	0.27	0.08
Eriosoma sp. 2	0.44	0.19
Eriosoma sp. 3	0.56	0.27
Eriosoma sp. 4	0.31	0.14
Formosaphis micheliae	0.32	0.14
Kaburagia rhusicola	0.10	0.03
Pemphigus borealis	0.21	0.07
Pemphigus bursarius	0.20	0.06
Pemphigus matsumurai	0.11	0.03
Pemphigus populitransversus	0.10	0.03
Pemphigus sinobursarius	0.14	0.04
Pemphigus sp. 1	0.28	0.13
Pemphigus sp. 2	0.12	0.03
Pemphigus tibetensis	0.42	0.21
Prociphilus kuwanai	0.75	0.33
Prociphilus ligustrifoliae	0.52	0.25
Prociphilus pini	0.78	0.45
Prociphilus sp.	0.21	0.06
Schlechtendalia chinensis	0.11	0.04
Tetraneura sp. 1	0.31	0.12
Tetraneura sp. 2	0.19	0.07
Tetraneura sp. 3	0.55	0.30
Tetraneura sp. 4	0.28	0.11
Tetraneura sp. 5	0.21	0.09
Thecabius beijingensis	0.16	0.05
Thecabius sp. 1	0.27	0.08
Thecabius sp. 2	0.23	0.08
Thecabius sp. 3	0.15	0.05
Mean	0.29	0.12

Table S3. The relative abundance of top ten bacterial genera in Eriosomatinae.

Species	*Buchnera*	*Regiella*	*Gluconobacter*	*Serratia*	*Pectobacterium*	*Acinetobacter*	*Carsonella*	*Hamiltonella*	*Sphingobacterium*	*Akkermansia*	Others
Chaetogeoica sp.	0.969234	0.012602	2.43×10^{-5}	0.000162	0.00198	0.001043	0	0	8.08×10^{-6}	6.47×10^{-5}	0.001859
Colophina arctica	0.853049	0.032229	2.43×10^{-5}	0.002829	2.43×10^{-5}	1.62×10^{-5}	0	0	0	0	0.107212
Epipemphigus imaicus	0.984512	0.00519	0.001407	8.08×10^{-5}	0.000437	0.001237	0	0	1.62×10^{-5}	0	0.001681
Epipemphigus niisimae	0.97853	0.001213	0.0027	1.62×10^{-5}	0.00114	0.001552	0.000137	0	2.43×10^{-5}	0	0.013378
Epipemphigus yunnanensis	0.968353	0.012416	0	0.006321	0.00308	4.04×10^{-5}	0.000267	0.000364	0	0	0.007736
Eriosoma lanigerum	0.929705	0.014073	3.23×10^{-5}	8.89×10^{-5}	0.033813	0.000162	0	0	0	0	0.002999
Eriosoma sp. 1	0.956996	0.021413	8.08×10^{-6}	0.000703	0.001948	0.000954	0.000194	0.00038	8.08×10^{-6}	0	0.006572
Eriosoma sp. 2	0.895666	0.074619	0	0.017848	0.001997	8.89×10^{-5}	0.000226	0.000396	0	1.62×10^{-5}	0.006903
Eriosoma sp. 3	0.845531	0.132118	2.43×10^{-5}	0.001892	1.62×10^{-5}	0.001843	0	0	0.000154	8.08×10^{-6}	0.003864
Eriosoma sp. 4	0.927297	0.061872	1.62×10^{-5}	0.004155	1.62×10^{-5}	4.04×10^{-5}	0	0	0	0	0.002999
Formosaphis micheliae	0.926197	0.005594	8.08×10^{-6}	0.000485	0.060174	1.62×10^{-5}	0.000137	0.000348	8.08×10^{-6}	0	0.006378
Kaburagia rhusicola	0.984496	0.001706	8.08×10^{-6}	2.43×10^{-5}	0.001495	6.47×10^{-5}	0	0.001827	0	0	0.008973
Pemphigus borealis	0.965216	0.000251	0.001237	0.000137	0.005949	0.001277	0	0	0	8.08×10^{-6}	0.019974
Pemphigus bursarius	0.966857	0.017412	0	0.000445	0.003339	0	0.000186	0.000461	0.000614	8.08×10^{-6}	0.005796
Pemphigus matsumurai	0.985999	0.004923	3.23×10^{-5}	0.001463	8.08×10^{-6}	0.000113	0	0.001843	0	8.08×10^{-6}	0.002409
Pemphigus populitransversus	0.98507	0.001706	8.08×10^{-6}	3.23×10^{-5}	0.007089	8.89×10^{-5}	0	0	0	0	0.004486
Pemphigus sinobursarius	0.980114	0.005085	8.08×10^{-6}	0.002255	0.006629	6.47×10^{-5}	0	0	8.08×10^{-6}	4.85×10^{-5}	0.003492
Pemphigus sp. 1	0.928226	0.068007	8.08×10^{-6}	8.08×10^{-6}	0.001964	1.62×10^{-5}	8.08×10^{-6}	8.08×10^{-6}	0	0	0.000687
Pemphigus sp. 2	0.983073	0.006224	1.62×10^{-5}	0.00527	0.001989	0	0	0	0	0	0.001843
Pemphigus tibetensis	0.879943	0.00869	1.62×10^{-5}	0	0.00114	4.85×10^{-5}	0	0	0	0	0.108562
Prociphilus kuwanai	0.807862	0.000186	0.11376	0.000121	0.000226	0.037419	6.47×10^{-5}	8.08×10^{-6}	0.009288	0	0.019724
Prociphilus ligustrifoliae	0.861351	0.056068	0	0.076026	4.85×10^{-5}	8.08×10^{-6}	0	0	0	0	0.005561
Prociphilus pini	0.687231	0.005658	0.00291	0.000348	0.001892	0.001892	0.014122	0.000234	3.23×10^{-5}	0	0.281599
Prociphilus sp.	0.970147	0.004551	0.002021	0.00582	0.00038	0.002077	0	0	8.08×10^{-6}	0	0.007445
Schlechtendalia chinensis	0.981076	0.013685	1.62×10^{-5}	0.002183	0.001722	4.85×10^{-5}	8.08×10^{-6}	0	0	0	0.000412
Tetraneura sp. 1	0.936609	0.043457	0	0.000647	0.007469	7.28×10^{-5}	0.000154	0.000461	0	1.62×10^{-5}	0.006426
Tetraneura sp. 2	0.966114	0.014219	0	0.000291	0.001293	8.08×10^{-6}	0.000218	0.000372	1.62×10^{-5}	8.08×10^{-6}	0.016507
Tetraneura sp. 3	0.823552	0.15581	1.62×10^{-5}	0.000509	0.001997	8.08×10^{-6}	0.000283	0.000469	0	8.08×10^{-6}	0.016604
Tetraneura sp. 4	0.942841	0.042705	8.08×10^{-6}	0.002692	0.00557	0.00017	0	0	0	8.08×10^{-6}	0.003436
Tetraneura sp. 5	0.954708	0.037985	8.08×10^{-6}	0.001673	0	5.66×10^{-5}	0	8.08×10^{-6}	0	0	0.0046
Thecabius beijingensis	0.97448	0.013209	0.000501	2.43×10^{-5}	0.001665	5.66×10^{-5}	0	0	0	0	0.007526

Thecabius sp. 1	0.957529	0.005351	0	0.000437	0.001722	5.66×10^{-5}	0.000154	0.012441	0	0.00675	0.007057
Thecabius sp. 2	0.958305	0.006103	0.003533	0.001997	3.23×10^{-5}	0.001827	0.000291	8.08×10^{-6}	4.85×10^{-5}	0	0.024695
Thecabius sp. 3	0.974537	0.017566	0	0.002344	0.000291	0.000194	0	8.08×10^{-6}	1.62×10^{-5}	0	0.002967

Table S4. OTUs and reads number of facultative symbionts.

**Sample**	***Arsenophonus***	***Hamiltonella***	***Regiella***					***Rickettsia***	***Serratia***		***Spiroplasma***
OTU_name	OTU_58	OTU_32	OTU_19	OTU_25	OTU_42	OTU_701	OTU_993	OTU_733	OTU_33	OTU_22	OTU_162
13509a	0	0	95	0	0	0	0	0	0	1	0
13509b	0	0	59	0	0	0	0	0	0	0	0
13509c	0	0	66	0	0	0	0	0	0	0	0
14139a	0	0	3930	1	3	5	0	0	0	0	0
14139b	0	1	4928	1	5	3	0	0	0	1	0
14139c	0	0	3939	0	4	0	0	0	0	0	0
14166a	0	0	113	0	0	0	0	0	0	0	15
14166b	0	0	95	1	0	0	0	0	0	1	2
14166c	0	0	73	0	0	0	0	0	0	0	8
14167a	0	0	378	1	1	4	0	0	0	273	0
14167b	0	0	455	0	0	6	0	2	0	375	0
14167c	0	0	326	0	0	6	0	0	0	339	1
15300a	0	0	503	1	2	0	0	0	0	0	0
15300b	0	0	654	1	0	0	0	0	0	0	0
15300c	0	0	485	0	0	0	0	0	1	0	0
15412a	30	0	801	0	1	3	0	0	0	0	0
15412b	12	0	795	1	0	1	0	0	0	1	0
15412c	9	0	741	0	2	0	1	0	0	1	0
15429a	0	0	565	0	0	4	0	0	0	0	1
15429b	0	0	461	0	0	3	0	0	0	0	0
15429c	0	0	494	1	0	2	1	0	0	0	0
15699a	0	131	99	0	0	0	0	0	0	1	0
15699b	0	95	104	1	1	0	0	0	0	0	0
15699c	0	69	64	0	0	0	0	0	0	1	1
15703a	0	0	568	294	76	0	0	0	149	0	0
15703b	0	0	413	163	50	0	0	0	96	0	0
15703c	0	0	364	150	43	0	0	0	97	0	2
15739a	0	0	731	0	0	1	4	0	0	0	0
15739b	0	0	859	1	0	0	1	0	0	1	0
15739c	0	0	764	0	1	0	3	1	0	0	0

16169a	9	32	388	73	0	0	3	0	43	0	0
16169b	1	9	127	24	0	0	0	0	8	0	0
16169c	2	7	311	59	1	0	0	0	17	0	0
17573a	12	40	968	109	3	0	4	0	53	0	0
17573b	2	18	812	30	0	0	1	0	5	2	0
17573c	9	27	1158	80	3	1	2	0	27	0	0
18074a	5	26	334	82	0	0	2	0	37	1	3
18074b	2	12	129	28	0	0	0	1	11	2	0
18074c	6	13	219	51	0	0	0	0	22	0	1
18234a	5	32	741	106	2	8	0	0	54	290	0
18234b	2	13	504	22	0	6	0	0	15	319	0
18234c	4	21	719	68	5	6	0	0	20	406	0
22383a	7	30	1243	91	4	0	0	0	43	1	1
22383b	3	15	897	20	0	0	0	0	15	2	0
22383c	1	24	1305	67	1	0	1	0	28	1	0
22389a	113	31	1943	77	8	0	3	0	47	0	0
22389b	161	16	1789	27	9	0	2	0	11	0	0
22389c	219	31	3478	86	11	0	1	0	31	3	0
22400a	5	29	986	78	0	3	3	0	32	0	0
22400b	1	14	500	18	0	1	2	0	9	1	0
22400c	2	20	949	50	1	0	3	0	22	0	2
22912a	7	546	326	94	2	0	1	0	35	0	0
22912b	2	745	144	23	0	0	0	0	10	0	0
22912c	3	788	251	61	0	0	0	0	20	0	0
22920a	2	39	1846	2533	116	4	2	1	984	0	75
22920b	1	10	1375	2476	29	2	0	0	1132	0	15
22920c	2	22	1732	2623	66	2	0	2	958	0	89
22926a	3	25	7372	96	5	1	33	0	42	0	1
22926b	3	24	7283	35	0	2	13	0	6	0	0
22926c	4	25	9927	65	9	1	25	0	39	1	0
22942a	0	1	458	1	14	5	1	0	0	135	0
22942b	0	0	230	0	4	4	0	0	0	90	0
22942c	0	0	351	0	6	0	0	0	0	117	0
22956a	0	0	993	0	18	8	0	0	0	129	0

22956b	0	1	1092	0	18	6	1	0	0	127	0
22956c	0	0	829	0	9	3	0	0	0	122	0
22964a	0	0	323	0	17	2	0	0	1	114	0
22964b	0	0	185	0	6	5	1	0	0	108	1
22964c	0	0	307	0	11	2	0	0	0	152	0
22989a	0	0	1870	0	20	64	0	0	1	2019	0
22989b	0	0	2142	0	11	84	0	0	0	3117	0
22989c	0	0	4259	0	16	97	0	0	1	6391	0
23044a	0	0	2962	1	16	5	0	0	0	77	0
23044b	0	0	3920	0	7	3	0	0	0	86	0
23044c	0	0	14439	1	10	1	0	0	0	119	0
23047a	24	0	2225	1	29	1	3	0	0	130	0
23047b	20	0	2637	0	28	6	3	0	0	167	0
23047c	6	0	2332	0	9	4	2	0	0	157	0
23081a	0	0	2171	0	18	13	3	0	0	88	0
23081b	0	1	1991	0	8	9	4	0	0	67	0
23081c	0	0	1913	0	2	8	2	0	0	112	0
23129a	0	149	355	0	15	5	2	0	0	90	0
23129b	0	103	201	0	11	0	1	0	0	74	0
23129c	0	80	254	0	11	0	0	0	0	81	0
23142a	0	0	2386	397	348	7	0	0	196	131	0
23142b	0	0	2314	287	363	6	0	1	131	107	0
23142c	0	0	3816	170	575	2	0	0	91	112	0
23540a	0	0	1590	0	31	5	8	0	2	155	0
23540b	0	0	1825	1	13	4	1	0	1	146	0
23540c	0	0	1830	0	12	6	6	0	2	154	0
24365a	0	0	16	0	1	0	0	0	0	0	0
24365b	0	0	12	0	0	0	0	0	1	0	0
24365c	0	1	8	0	0	0	0	0	0	0	0
25471a	0	0	323	0	1	0	0	0	1	0	0
25471b	0	0	316	0	1	1	0	0	0	0	0
25471c	0	0	280	0	0	0	0	0	1	1	0
25483a	0	0	15	0	2	0	0	0	1	0	1
25483b	0	0	13	1	0	0	0	0	0	2	0

25483c	0	0	12	0	0	0	0	0	0	0	0
Y8936a	0	0	252	0	1	5	0	0	0	258	0
Y8936b	0	0	256	0	0	2	1	0	0	350	0
Y8936c	0	0	241	0	0	6	0	0	0	333	0

Table S5. Infection pattern of facultative symbionts in Eriosomatinae.

Secondary symbiont infection type	Species	Infection frequency
Regiella–Serratia	*Chaetogeoica* sp.	7/34
Regiella–Serratia	*Colophina arctica*	
Regiella–Serratia	*Epipemphigus imaicus*	
Regiella–Serratia	*Epipemphigus niisimae*	
Regiella–Serratia	*Eriosoma* sp. 3	
Regiella–Serratia	*Prociphilus ligustrifoliae*	
Regiella–Serratia	*Prociphilus* sp.	
Regiella–Spiroplasma	*Pemphigus tibetensis*	1/34
Arsenophonus–Regiella–Serratia	*Eriosoma lanigerum*	2/34
Arsenophonus–Regiella–Serratia	*Tetraneura* sp. 4	
Hamiltonella–Regiella–Serratia	*Pemphigus matsumurai*	6/34
Hamiltonella–Regiella–Serratia	*Pemphigus* sp. 1	
Hamiltonella–Regiella–Serratia	*Thecabius* sp. 2	
Hamiltonella–Regiella–Serratia	*Thecabius* sp. 3	
Hamiltonella–Regiella–Serratia	*Tetraneura* sp. 5	
Hamiltonella–Regiella–Serratia	*Prociphilus kuwanai*	
Regiella–Rickettsia–Serratia	*Eriosoma* sp. 4	2/34
Regiella–Rickettsia–Serratia	*Thecabius beijingensis*	
Regiella–Serratia–Spiroplasma	*Pemphigus borealis*	4/34
Regiella–Serratia–Spiroplasma	*Pemphigus populitransversus*	
Regiella–Serratia–Spiroplasma	*Pemphigus sinobursarius*	
Regiella–Serratia–Spiroplasma	*Schlechtendalia chinensis*	
Arsenophonus–Hamiltonella–Regiella–Serratia	*Epipemphigus yunnanensis*	5/34
Arsenophonus–Hamiltonella–Regiella–Serratia	*Pemphigus bursarius*	
Arsenophonus–Hamiltonella–Regiella–Serratia	*Prociphilus pini*	
Arsenophonus–Hamiltonella–Regiella–Serratia	*Tetraneura* sp. 1	
Arsenophonus–Hamiltonella–Regiella–Serratia	*Thecabius* sp. 1	
Hamiltonella–Regiella–Serratia–Spiroplasma	*Kaburagia rhusicola*	1/34
Regiella–Rickettsia–Serratia–Spiroplasma	*Pemphigus* sp. 2	1/34
Arsenophonus–Hamiltonella–Regiella–Serratia–Spiroplasma	*Eriosoma* sp. 1	3/34
Arsenophonus–Hamiltonella–Regiella–Serratia–Spiroplasma	*Tetraneura* sp. 2	

Arsenophonus–Hamiltonella–Regiella–Serratia–Spiroplasma	*Tetraneura* sp. 3	
Arsenophonus–Hamiltonella–Regiella–Rickettsia–Serratia–Spiroplasma	*Eriosoma* sp. 2	2/34
Arsenophonus–Hamiltonella–Regiella–Rickettsia–Serratia–Spiroplasma	*Formosaphis micheliae*	

Table S6. Spearman correlation coefficients of symbionts in Eriosomatinae.

**Symbionts**	**Symbionts**	*ρ*	***p* value**
Arsenophonus	*Buchnera*	−0.34	0.05
Arsenophonus	*Hamiltonella*	0.54	**<0.001**
Arsenophonus	*Regiellaa*	0.29	0.09
Arsenophonus	*Rickettsia*	0.02	0.91
Arsenophonus	*Serratia*	0.03	0.86
Arsenophonus	*Spiroplasma*	0.09	0.62
Buchnera	*Hamiltonella*	−0.11	0.54
Buchnera	*Regiella*	−0.51	**0.002**
Buchnera	*Rickettsia*	−0.06	0.75
Buchnera	*Serratia*	−0.07	0.70
Buchnera	*Spiroplasma*	0.05	0.76
Hamiltonella	*Regiella*	0.03	0.86
Hamiltonella	*Rickettsia*	−0.05	0.80
Hamiltonella	*Serratia*	−0.03	0.87
Hamiltonella	*Spiroplasma*	0.18	0.30
Regiella	*Rickettsia*	0.17	0.34
Regiella	*Serratia*	0.40	**0.02**
Regiella	*Spiroplasma*	−0.08	0.64
Rickettsia	*Serratia*	0.24	0.17
Rickettsia	*Spiroplasma*	0.17	0.34
Serratia	*Spiroplasma*	−0.09	0.62

Significant *p* values ($p < 0.05$) are in bold.

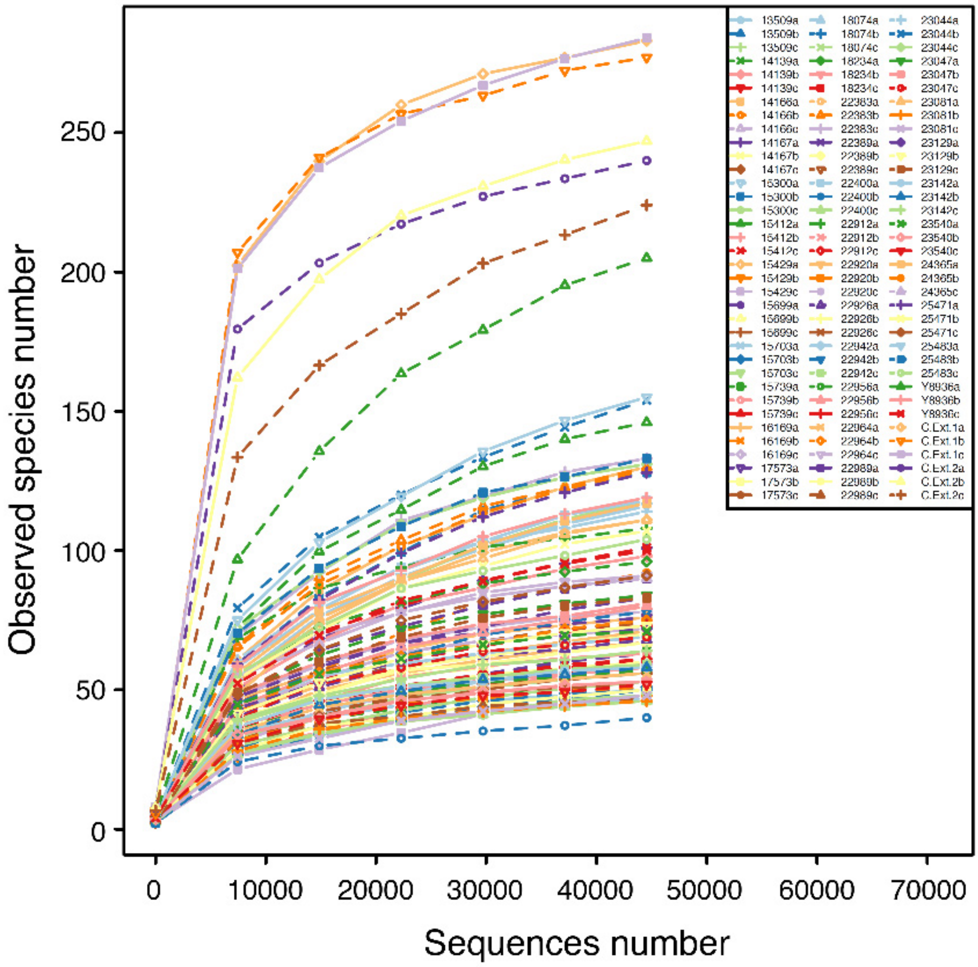

Figure S1. Rarefaction curve for each sample of Eriosomatinae based on the index of observed species.
